# Prediction and design optimization of mechanical properties for rubber fertilizer hose reinforced with helically wrapped nylon

**DOI:** 10.1038/s41598-024-64233-y

**Published:** 2024-06-10

**Authors:** Mengfan Wang, Lixin Zhang, Changxin Fu

**Affiliations:** 1 College of Mechanical and Electrical Engineering, Shihezi University, Shihezi, 832003 China; 2 Xinjiang Production and Construction Corps Key Laboratory of Modern Agricultural Machinery, Shihezi, 832003 China; 3 Key Laboratory of Northwest Agricultural Equipment, Ministry of Agriculture and Rural Affairs, Shihezi, 832003 China; 4 Bingtuan Energy Development Institute, Shihezi University, Shihezi, 832003 China

**Keywords:** Prediction of mechanical properties, Multi-objective optimization design, CPO-GRNN, Fiber-reinforced composite material fertilizer hoses, Engineering, Mechanical engineering

## Abstract

Predicting and optimizing the mechanical performance of the helically wound nylon-reinforced rubber fertilizer hose (HWNR hose) is crucial for enhancing the performance of hose pumps. This study aims to enhance the service life of HWNR hoses and the efficiency of liquid fertilizer transport. First, a finite element simulation model and a mathematical model were established to analyze the influence of fiber layer arrangement on the maximum shear strain on the coaxial surface (MSS) and the reaction force on the extrusion roller (RF). For the first time, the Crested Porcupine Optimizer algorithm was used to improve the Generalized Regression Neural Network (CPO-GRNN) method to establish a surrogate model for predicting the mechanical properties of HWNR hoses, and it was compared with Response Surface Methodology (RSM). Results showed CPO-GRNN's superiority in handling complex nonlinear problems. Finally, the Non-dominated Sorting Genetic Algorithm II (NSGA-II) was employed for optimization design. Compared to the original HWNR hose with an MSS of 0.906 and an RF of 30,376N, the optimized design reduced the MSS by 7.99% and increased the RF by 2.46%, significantly enhancing their service life and liquid fertilizer transport capacity. However, further research on fatigue damage is needed.

## Introduction

**Figure 1:**
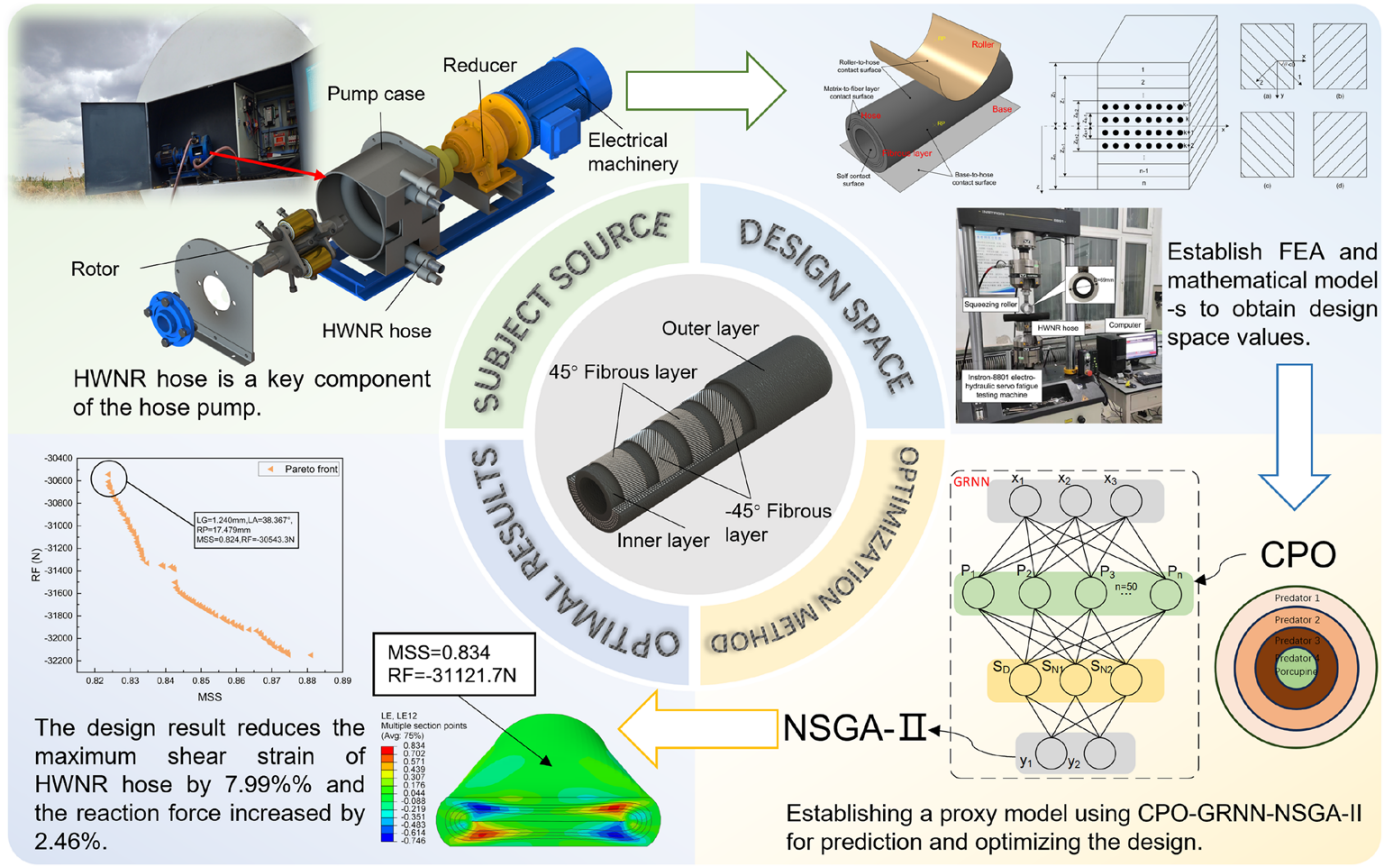
Overview of the Study. This figure provides an overview of the study, with arrows indicating the topic's source, modeling, optimization design method, and optimization results.

The large-scale cultivation in Northwest China necessitates extensive fertilizer application. Liquid fertilizer, with its high efficiency, low cost, and ease of application, has been widely adopted. The hose pump, as a novel fertilizer delivery mechanism, holds great potential due to its components' corrosion resistance and ability to convey high-viscosity fluids. Comprising a casing, hose, rotor, gearbox, and motor, as illustrated in Fig. 1, the hose stands out as the pivotal component of the pump. During operation, the squeezing rollers compress the hose, creating a sealed cavity. As the rotor rotates, the hose rebounds, generating negative pressure and drawing fluid into the hose. Subsequently, the rotating rotor propels the liquid within the hose towards the pump outlet^1^. Hence, the deformation and rebound capability of the hose directly impact the efficiency of liquid fertilizer transport. Typically, the HWNR hose is employed for hose pump compression. The failure of HWNR hoses can be categorized into two types: normal and abnormal failure. Normal failures include fatigue failure and frictional wear, while abnormal failures encompass tear damage and sudden bursting. Fatigue failure, characterized by the detachment of fiber layers from the matrix, is the primary cause of hose failure. Prolonged exposure to alternating loads may lead to fatigue failure forms such as the ellipticalization of the hose cross-section^2^. In order to enhance the efficiency of liquid fertilizer transport through HWNR hoses and improve their resistance to fatigue failure, CPO-GRNN was employed to predict the mechanical properties of HWNR hoses and conduct multi-objective optimization design.

Traditional optimization design generally consists of three aspects: design variables, experimental design, and optimization design. First, the variables are designed. Traditional design variable manipulation involves single-factor experiments, undoubtedly increasing experimental and computational costs. In this study, mathematical model analysis replaces this process. HWNR hoses are made of fiber reinforced composites (FRCs), which exhibit excellent modulus, strength, and fatigue performance and are widely used in fields such as automotive and aerospace^3,4^. According to the mechanics of composite materials, research on FRCs' constitutive behavior can be divided into macro-mechanics and micro-mechanics. For macro-mechanics, there are laminate theory^5^ and Knapp's theories^6^. Both methods are mature, but Knapp's theories often involve complex mathematical derivations and computations, making them less suitable for simple engineering design and analysis. Therefore, this paper chooses laminate theory. For micro-mechanics, the method of selecting Representative Volume Elements (RVEs) is generally adopted. However, selecting RVEs inevitably leads to homogenization characteristics, which affect the estimation of stress and strain distributions. To mitigate this effect, scholars have proposed many empirical models, such as mixture rules, the Halpin–Tsai model, the Cox–Krenchel model, etc.^7,8^. This paper uses the commonly used Halpin–Tsai model to illustrate the influence of various factors on the material modulus. This model considers fiber length, fiber volume fraction, and the interface effect between fibers and the matrix, providing a simple yet effective method for estimating the modulus of composite materials^9^, with subsequent improvements^10–12^. In this study, the laminate theory and Halpin–Tsai formula are used to construct the mechanical model of HWNR hoses. Combining actual production conditions, the effects of the laying angle, laying position, and laying gap of the nylon reinforcement layer on the mechanical properties of the material are determined. At the same time, the optimal level range of each factor is also determined, avoiding single-factor experiments using traditional optimization methods.

In terms of experimental design, this paper establishes models and obtains sample data through finite element analysis (FEA). Nikola Korunović et al. analyzed several common material models for tire research, namely linear models, Yeoh models, and Marlow models^13^, providing guidance for the selection of fiber composite rubber materials. Yue Yu et al. developed a reliable numerical model with acceptable complexity for non-woven fabric composite rubber hoses and conducted experimental verification^14^. Subsequently, an improved neural network is established as a surrogate model. Traditional optimization design methods such as orthogonal design and response surface design generally rely on least squares regression functions, exhibiting poor predictive capabilities for highly nonlinear problems. Neural networks, on the other hand, offer better solutions to this issue. In recent years, machine learning and neural network methods have been increasingly applied in the field of optimization design^15^. Improving neural networks through optimization algorithms is a common approach. This paper employs the Crested Porcupine Optimizer (CPO) algorithm to enhance the Generalized Regression Neural Network (GRNN). Mohamed Abdel-Basset et al. simulated the defense strategies of crested porcupines, developed the CPO algorithm, and demonstrated its excellent performance^16^. Due to its low sample learning and high accuracy^17^, GRNN is widely used in regression analysis. Many improved algorithms based on the GRNN algorithm have been proposed, such as combining the variable fidelity surrogate method to develop a surrogate model^18^, improving the Pearson correlation coefficient between GRNN inputs and outputs to determine weights between input and pattern layers^19^, and using the Black Widow Optimization algorithm (BWO) to improve GRNN^20^. The mechanical performance of HWNR hoses is influenced by multiple factors and exhibits strong nonlinearity. In this paper, the CPO-GRNN method is employed for the first time to construct a surrogate model within the domain of the objective function, significantly enhancing prediction accuracy. By utilizing this model for predicting the mechanical performance of HWNR hoses, highly accurate results are obtained.

In terms of design optimization, there are many multi-objective optimization algorithms currently used, such as Non-dominated Sorting Genetic Algorithm II (NSGA-II), Multi-Objective Particle Swarm Optimization (MOPSO) and Multi-Objective Seagull Optimization Algorithm (MOSOA)^21–24^. Among them, NSGA-II has become one of the most commonly used algorithms due to its ability to maintain solution distribution and converge better to the true Pareto optimal front^25^. Kai Liu et al. developed a new type of bamboo foam-filled tube (BFFT), established a finite element analysis model, and conducted multi-objective optimization design using a response surface combined with NSGA-II method^26^. Heng-Yi Li et al. used NSGA-II for the optimization design of desiccant wheels^27^. Kui Xu et al. designed a multi-objective optimization strategy based on NSGA-II, obtaining the optimal performance of high-power proton exchange membrane fuel cell systems under different operating conditions^28^. Mengtian Fan et al. applied NSGA-II to the multi-objective optimization of recycled aggregate concrete mix proportions^29^. This paper employs the CPO-GRNN-NSGA-II method for multi-objective optimization design and conducts FEA to validate the accuracy of the optimization results. The obtained results are highly satisfactory, significantly enhancing the performance of hose pumps.

Following the outlined process, this paper predicts and optimizes the mechanical properties of the HWNR hose to enhance the service life of the hose pump and improve the efficiency of transporting liquid fertilizer. Additionally, based on the theories of macroscopic and microscopic material mechanics, as well as FEA, the mechanical characteristics of the HWNR hose under squeezing roller compression are theoretically, experimentally, and numerically analyzed, as depicted in Fig. 1. The remainder of the paper is organized as follows: Section “Simplified simulation model and numerical calculation method for HWNR hoses” introduces the simulation model of the HWNR hose and its experimental validation. Section “Selection and analysis of design variables for HWNR hose” focuses on determining the primary cause of fatigue failure in HWNR hoses and optimizing variable design in conjunction with theoretical formulas. In Section “Parameter optimization and analysis”, CPO-GRNN was used to predict the mechanical properties of HWNR hoses, and optimization results were obtained by combining NSGA-II. Finally, Section “Concluding remarks” summarizes the conclusions.

## Simplified simulation model and numerical calculation method for HWNR hoses

**Figure 2:**
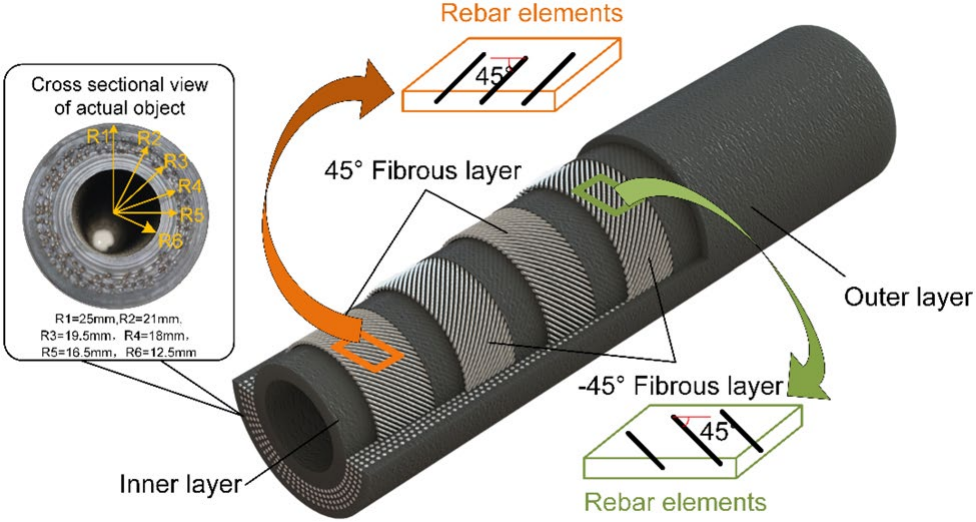
HWNR Hose Model and Partial Dimensions.

The materials constituting HWNR hoses mainly include two types: nylon and natural rubber (NR). In the middle fiber layer of the hose, nylon threads are laid out in a ± 45° angle, as shown in Fig. 2. There are a total of 4 layers of fiber, with a cross-sectional radius of 0.5mm for the nylon threads and a distance of 1.5mm between them. The bonding medium for the fiber layers is NR. NR is used for the inner and outer layers of the hose. NR material has high elasticity, tensile strength, and excellent tear resistance.

The use of NR material for the inner layer can enhance the service life of the hose due to its ability to withstand significant deformation. This study selected HWNR hoses with an outer diameter of 50mm and an inner diameter of 25mm, with specific dimensions detailed in Table 1.

**Table 1:** Dimensional Parameters of HWNR Hose.

Outer diameter (mm)	Inner diameter (mm)	Nylon diameter (mm)
50	25	1
Fiber Layer Inter-Layer Height (mm)	Fiber Layer Spacing (mm)	Fiber Layer Laying Angle (°)
1.5	1.5	± 45

### Modeling of the HWNR hose

**Figure 3:**
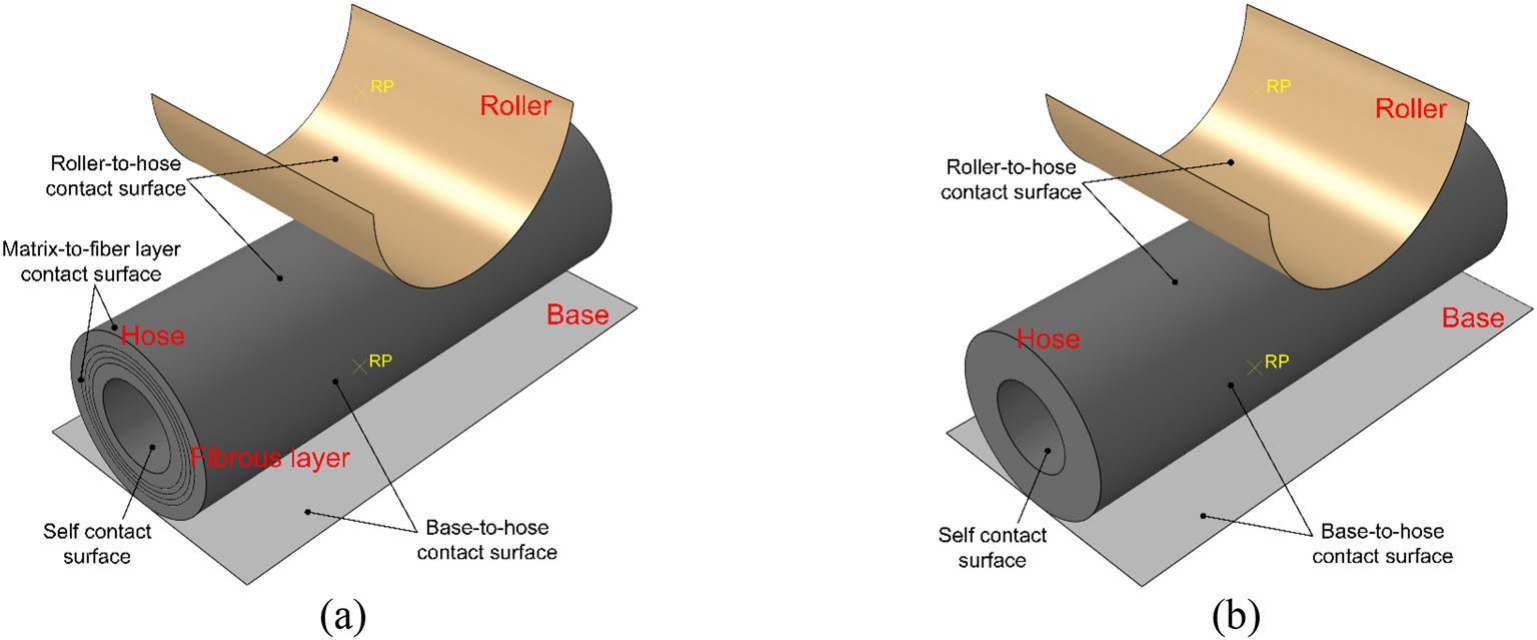
Finite Element Models. (**a**) Finite Element Model of Hose with Fiber Layer. (**b**) Finite Element Model of Pure Rubber Hose.

To simplify calculations and reduce computational complexity, the rolling motion of the extrusion roller over the hose circumference is simplified as uniform downward motion of the extrusion roller pressing the composite hose. We utilized Abaqus 2023 for simulation modeling. By calculating the actual pressing time of the extrusion roller under working conditions, the simulation time was set to 0.15s, and the pressing distance was set to 35 mm. The model of the extrusion roller pressing the hose consists of eight parts: the extrusion roller, the casing, the hose, the fiber layer, the contact surface between the hose and the extrusion roller, the contact surface between the fiber layer and the hose, the contact surface between the hose and the casing, and the self-contact surface of the inner wall of the hose, as shown in Fig. 3a. To reduce the number of mesh elements and simulation time, the dispersion method was employed to arrange reinforcement film for model establishment. The extrusion roller and casing were modeled using analytical rigid bodies. Shell elements were utilized for the fiber layer, while solid elements were employed for the hose. At the same time, to determine the stress characteristics of pure rubber hoses for optimizing the fiber laying angle, a model of the extrusion roller pressing rubber hoses of the same material was established. The model is essentially similar to the composite hose model, as shown in Fig. 3b.

### Material property settings

As mentioned earlier, the optimized hose consists of two materials: Vulcanized natural rubber matrix and nylon fibers. The properties of these two materials differ significantly. To reduce modeling complexity and improve simulation convergence speed, we used a reinforced concrete model to simulate the fiber winding layer. Additionally, a cylindrical coordinate system was defined to determine the fiber direction. Nylon is treated as an isotropic material, and the material parameters for the fiber layer are defined as shown in Table 2.

**Table 2:** Nylon Material Parameters.

Density (tonne/mm^3^)	Young's Modulus (MPa)	Poisson's ratio
1.15 × 10^–9^	3000	0.3

**Figure 4:**
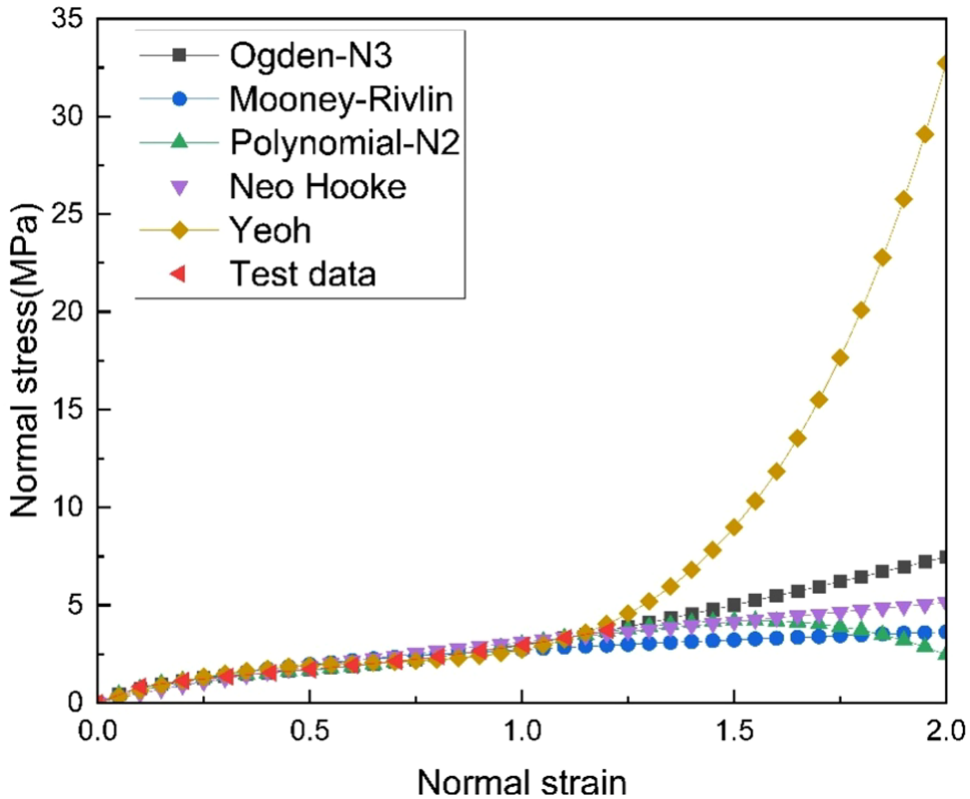
Vulcanized Natural Rubber Material Experimental Data and Constitutive Model Fitting Curve.

Vulcanized natural rubber is a hyperelastic material. The manufacturer commissioned uniaxial tensile tests on rubber samples, and the experimental data is shown in Fig. 4.

Compared to other constitutive models, as shown in Fig. 4, the Yeoh model requires fewer parameters and can yield reasonable results based solely on uniaxial tensile tests. Therefore, the vulcanized natural rubber material is set to the Yeoh model. The expression for the strain energy density of the Yeoh model for polynomial hyperelastic materials is as follows^30^.

1 $$ W = \mathop \sum \limits_{i = 0}^{3} C_{i0} (\overline{I}_{1} - 3)^{i} + \mathop \sum \limits_{i = 1}^{3} D_{i} (J - 1)^{2i} $$

In the equation, *C*_*i*0_ and *D*_*i*_ are input parameters determined experimentally. *I*_1_ is the first strain tensor invariant. *J* is the ratio of deformed volume to undeformed volume, which equals 1 for incompressible materials. The material parameters are listed in Table 3.

**Table 3:** Vulcanized Natural Rubber Material Parameters.

Density (tonne/mm^3^)	*C*_10_ (Mpa)	*C*_20_ (Mpa)	*C*_30_ (Mpa)
1.12 × 10^–9^	1.18	− 0.29	6.23 × 10^–2^

### Analysis model and interaction setup

The compression of composite hoses by extrusion rollers presents a typical nonlinear problem, incorporating material nonlinearity, geometric nonlinearity, and contact nonlinearity. Implicit algorithms often encounter convergence challenges in such scenarios. Hence, this simulation employs an explicit dynamic algorithm. In contrast to implicit algorithms, explicit algorithms solely necessitate previous displacements, velocities, and displacements at the prior time step for solving, without requiring iteration within each incremental step. Furthermore, they obviate the need for forming tangent stiffness matrices, rendering convergence comparatively simpler.

Hose pumps feature two roller configurations: shoe-type and roller-type. The interaction between the rollers and the hose varies depending on whether it entails sliding friction (shoe-type) or rolling friction (roller-type). To minimize friction, lubricating oil is introduced into the housing of shoe-type hose pumps. The friction coefficients for both roller configurations are similar. Interactions within the model are categorized into three types: surface-to-surface contact, self-contact, and embedded regions. Surface-to-surface contact and self-contact are employed to delineate interactions among the extrusion rollers, composite hose, and housing. Tangential behavior is governed by penalty functions with a friction coefficient of 0.1, while normal behavior is characterized as hard contact. Embedded regions are utilized to define the reinforcement structure within the rubber hose matrix.

### Meshing

In order to enhance simulation accuracy, the simulation employs fully integrated C3D8R elements to simulate the rubber matrix. These elements accurately depict hyperelastic materials such as rubber and resist shear locking under bending loads. SFM3D4R elements are utilized to simulate the fiber layers. The reinforcement method essentially involves constructing overlapping elements (reinforcement elements) embedded within the matrix elements, sharing nodes with the matrix elements, to simulate the composite reinforced material in finite element analysis. This approach significantly simplifies the modeling of composite hose materials.

Due to the "hourglass" problem associated with C3D8R elements, the mesh density is increased, with a mesh size set to 1.5mm, to minimize adverse effects. Additionally, mesh independence studies are conducted to verify mesh insensitivity. The force exerted on the extrusion roller in the y-direction when it is pressed down by 35 mm is used as a reference independent of the mesh. Mesh sizes of 1.26 mm, 1.5874 mm, 2 mm, and 2.52 mm are tested. When there is no fiber layer present, the extrusion roller force remains around 5600 N for all mesh sizes. However, with the fiber layer, the extrusion roller force is approximately 30000 N. The error between consecutive solutions is within 1%, indicating mesh independence has been achieved with a mesh size of 1.5 mm, meeting the requirements for computational accuracy and speed.

### Experimental setup and result comparison

**Figure 5:**
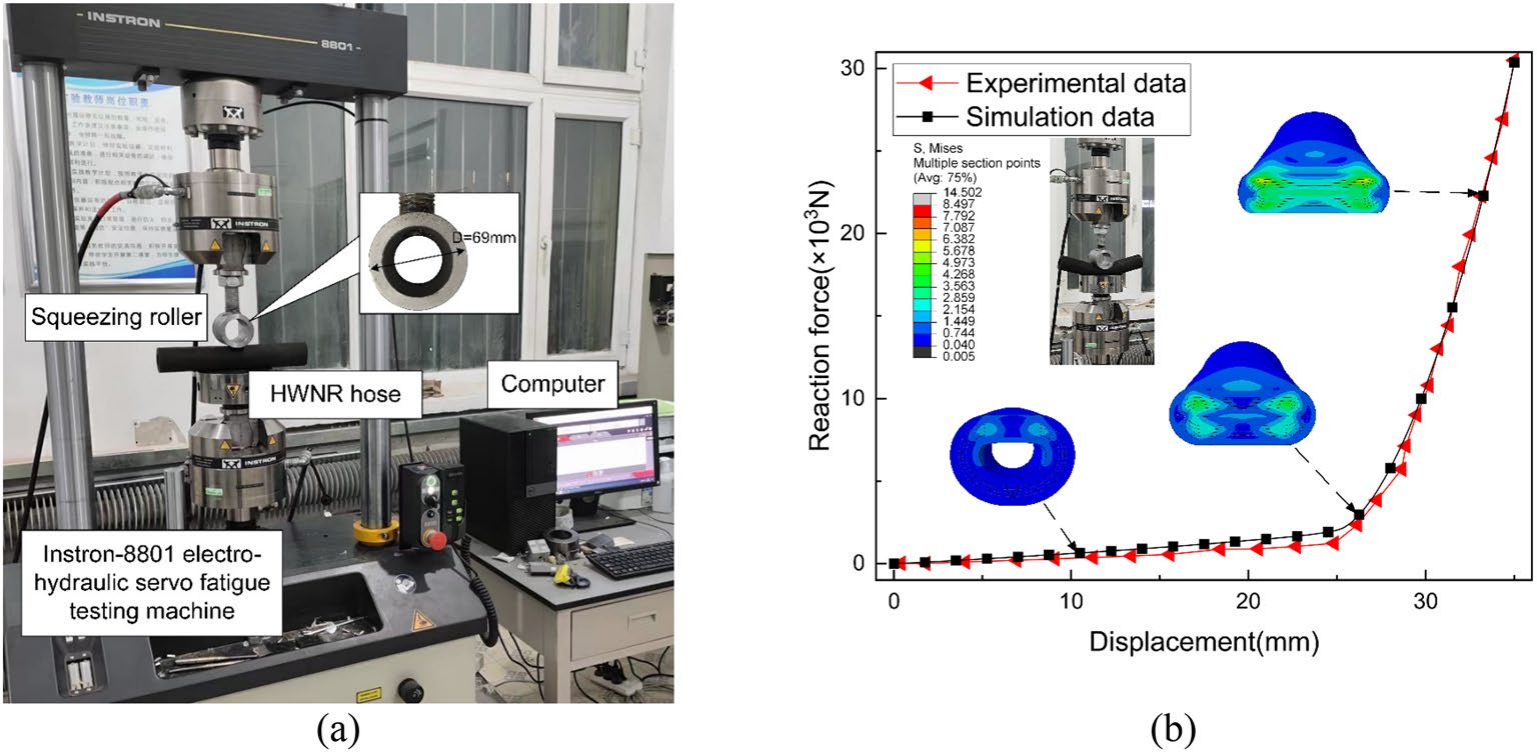
HWNR hose Extrusion Test. (**a**) Experimental Platform. (**b**) Comparison between Simulation Results and Experimental Results.

The experimental testing was conducted using an open test bench system. We employed an Instron-8801 electro-hydraulic servo fatigue testing machine for compressing the composite material hose, as depicted in Fig. 5a.

The experimental setup consisted of a metal extrusion roller with an outer diameter of 69mm, an HWNR hose with an outer diameter of 50mm and an inner diameter of 25mm, an Instron-8801 electro-hydraulic servo fatigue testing machine, and a computer. The extrusion roller was driven downward by a hydraulic cylinder during the upward stroke, with a motion range of [0, 35] mm. The force exerted on the hose was measured. The experimental results were then compared with the simulation results, as shown in Fig. 5b. To reduce experimental result errors caused by structural differences in various positions of the tube due to the manufacturing process, five independent repeated experiments were conducted according to Basel's formula, with an uncertainty of less than 0.02. The error between the experimental and simulation results was within 5%, validating the accuracy of the model.

## Selection and analysis of design variables for HWNR Hose

**Figure 6:**
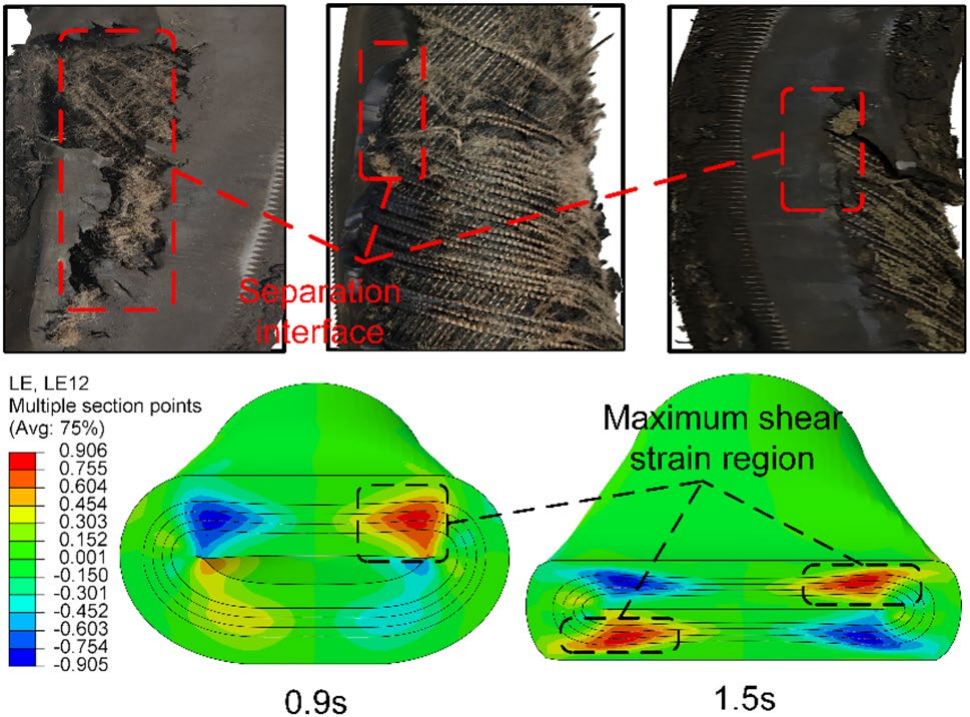
Contour Map of Axial Shear Strain in Extruded HWNR hose and Failure Location of HWNR hose.

From Fig. 6, it can be observed that the location of maximum shear strain on the coaxial surface of the fiber layer is essentially the same as the position where detachment between the NR matrix and the fiber layer occurs during actual hose usage. Moreover, this value is higher than the strain in other directions. Therefore, to reduce fatigue failure of the HWNR hose and enhance its service life, reducing the maximum shear strain on the coaxial surface of the fiber layer (MSS) is chosen as one of the optimization objectives. Meanwhile, according to the working principle of the hose pump, the ability of the hose to deform and rebound directly affects the efficiency of liquid fertilizer transport. Therefore, to increase the deformability and rebound capability of the HWNR hose, improve the efficiency of the hose pump, and reduce the accumulation of plastic deformation, increasing the stiffness of the HWNR hose is selected as another optimization objective. The absolute value of the increase in squeezing roller force (RF) is used to describe this objective.

### Optimization design of *fiber* layer radial position (RP)

**Figure 7:**
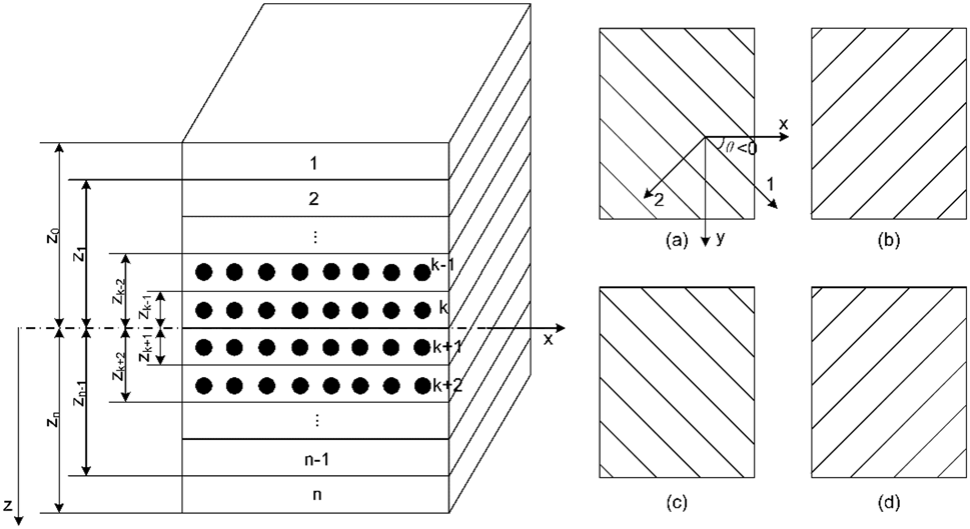
Laminate Model of HWNR hose Material.

To minimize the coaxial shear strain in HWNR hoses as much as possible, the optimization design of fiber layer placement is initiated. The HWNR hose material unit is simplified into laminate plate units, as illustrated in Fig. 7. The following assumptions are made for the laminate plate units under study:

1. Deformation between each layer of the laminate is continuous.
2. The total thickness adheres to the thin plate assumption.
3. The unit satisfies the hypothesis of normal deformation, with the length of the line remaining unchanged before and after deformation.

According to macroscopic mechanics of composite materials, the stress–strain formula for the k-th layer of a laminate plate is given by:

2 $$ \left. {\left[ {\begin{array}{*{20}c} {\sigma_{x} } \\ {\sigma_{y} } \\ {\tau_{xy} } \\ \end{array} } \right]_{k} = \left[ {\begin{array}{*{20}c} {\overline{Q}_{11} } & {\overline{Q}_{12} } & {\overline{Q}_{16} } \\ {\overline{Q}_{12} } & {\overline{Q}_{22} } & {\overline{Q}_{26} } \\ {\overline{Q}_{16} } & {\overline{Q}_{26} } & {\overline{Q}_{66} } \\ \end{array} } \right]_{k} \left\{ {\left[ {\begin{array}{*{20}c} {\varepsilon_{x}^{0} } \\ {\varepsilon_{y}^{0} } \\ {\gamma_{xy}^{0} } \\ \end{array} } \right]} \right. + z\left[ {\begin{array}{*{20}c} {\kappa_{x} } \\ {\kappa_{y} } \\ {\kappa_{xy} } \\ \end{array} } \right]} \right\} $$

In the equation, $\sigma_{x}$ and $\sigma_{y}$ represent normal stresses along the *x* and *y* directions, respectively. $\tau_{xy}$ denotes the shear stress in the *xy*-plane. The matrix $\overline{\user2{Q}}$ represents the transformation matrix for the two-dimensional stiffness matrix ***Q*** representing the principal directions, and it is related to the coordinate rotation angle $\theta$. $\kappa_{x} ,\kappa_{y}$ is called the curvature of the plate mid-plane, while $\kappa_{xy}$ represents the twisting curvature of the mid-plane, and $\varepsilon_{x}^{0} ,\varepsilon_{y}^{0} ,\gamma_{xy}^{0}$ denotes the mid-plane strain.

Integrating over each layer and summing them up yields the total internal force and internal moment of the laminated plate, as shown in Eq. (3).

3 $$\left[ {\begin{array}{*{20}c} {N_{x} } \\ {N_{y} } \\ {N_{xy} } \\ \end{array} } \right] = \mathop \sum \limits_{k = 1}^{n} \smallint_{{z_{k - 1} }}^{{z_{k} }} \left[ {\begin{array}{*{20}c} {\sigma_{x} } \\ {\sigma_{y} } \\ {\tau_{xy} } \\ \end{array} } \right]_{k} {\text{d}}z,{\kern 1pt} \left[ {\begin{array}{*{20}c} {M_{x} } \\ {M_{y} } \\ {M_{xy} } \\ \end{array} } \right] = \mathop \sum \limits_{k = 1}^{n} \smallint_{{z_{k - 1} }}^{{z_{k} }} \left[ {\begin{array}{*{20}c} {\sigma_{x} } \\ {\sigma_{y} } \\ {\tau_{xy} } \\ \end{array} } \right]_{k} z{\text{d}}z $$

where, *N*_*x*_, *N*_*y*_, *N*_*xy*_ represent the normal forces per unit width (or length) on the cross-section of the laminate, and *M*_*x*_, *M*_*y*_, *M*_*xy*_ represent the moments per unit width of the laminate cross-section. *n* is the number of layers in the laminate.

Substituting Eq. (2) into Eq. (3),

4 $$ \begin{array}{*{20}c} {\left[ {\begin{array}{*{20}c} {N_{x} } \\ {N_{y} } \\ {N_{xy} } \\ \end{array} } \right] = \left[ {\begin{array}{*{20}c} {A_{11} } & {A_{12} } & {A_{16} } \\ {A_{12} } & {A_{22} } & {A_{26} } \\ {A_{16} } & {A_{26} } & {A_{66} } \\ \end{array} } \right]\left[ {\begin{array}{*{20}c} {\varepsilon_{x}^{0} } \\ {\varepsilon_{y}^{0} } \\ {\gamma_{xy}^{0} } \\ \end{array} } \right] + \left[ {\begin{array}{*{20}c} {B_{11} } & {B_{12} } & {B_{16} } \\ {B_{12} } & {B_{22} } & {B_{26} } \\ {B_{16} } & {B_{26} } & {B_{66} } \\ \end{array} } \right]\left[ {\begin{array}{*{20}c} {\kappa_{x} } \\ {\kappa_{y} } \\ {\kappa_{xy} } \\ \end{array} } \right]} \\ {\left[ {\begin{array}{*{20}c} {M_{x} } \\ {M_{y} } \\ {M_{y} } \\ \end{array} } \right] = \left[ {\begin{array}{*{20}c} {B_{11} } & {B_{12} } & {B_{16} } \\ {B_{12} } & {B_{22} } & {B_{26} } \\ {B_{16} } & {B_{26} } & {B_{66} } \\ \end{array} } \right]\left[ {\begin{array}{*{20}c} {\varepsilon_{x}^{0} } \\ {\varepsilon_{y}^{0} } \\ {\gamma_{y} } \\ \end{array} } \right] + \left[ {\begin{array}{*{20}c} {D_{11} } & {D_{12} } & {D_{16} } \\ {D_{12} } & {D_{22} } & {D_{26} } \\ {D_{16} } & {D_{26} } & {D_{66} } \\ \end{array} } \right]\left[ {\begin{array}{*{20}c} {\kappa_{x} } \\ {\kappa_{y} } \\ {\kappa_{xy} } \\ \end{array} } \right]} \\ \end{array} $$

where, $$ \begin{array}{*{20}c} {A_{ij} = \mathop \sum \limits_{k = 1}^{n} \left( {\overline{Q}_{ij} } \right)_{k} \left( {z_{k} - z_{k - 1} } \right)} \\ {B_{ij} = \frac{1}{2}\mathop \sum \limits_{k = 1}^{n} \left( {\overline{Q}_{ij} } \right)_{k} \left( {z_{k}^{2} - z_{k - 1}^{2} } \right)} \\ {D_{ij} = \frac{1}{3}\mathop \sum \limits_{k = 1}^{n} \left( {\overline{Q}_{ij} } \right)_{k} \left( {z_{k}^{3} - z_{k = 1}^{3} } \right)} \\ \end{array}$$, *A*_*ij*_ is called the tensile stiffness, *D*_*ij*_ is called the bending stiffness, and *B*_*ij*_ is called the coupling stiffness.

**Figure 8:**
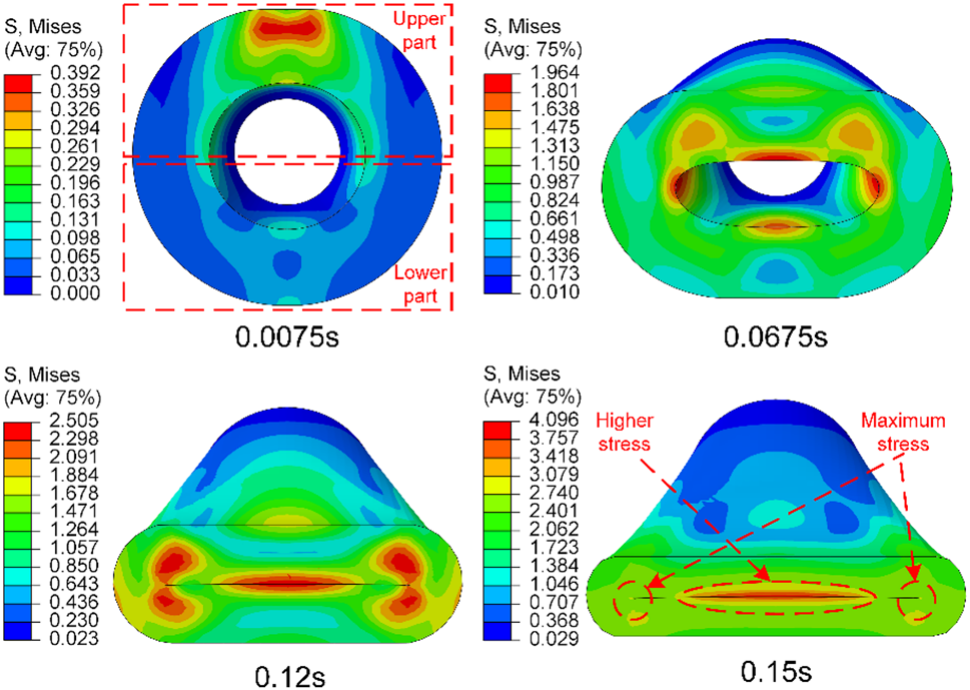
Von Mises stress contour plot during the extrusion process of the pure rubber hose.

From the above formulas, it can be observed that the stiffness of the HWNR hose is determined by each layer of the laminate, while the interaction between layers results in a coupling relationship between tension and bending. Stress analysis is conducted on the rubber hose compression process without fiber layers, and finite element simulation results reveal different stress states in the upper and lower parts of the hose on the same cross-section. During the compression process, under the combined action of bending and pressure, the middle of the upper half of the hose bends downwards, and as the extrusion roller continues downwards, the lowest point of the bent portion starts contacting the lower half until fully closed, as shown in Fig. 8. During compression, maximum stress and strain occur in the regions on both sides of the inner surface of the hose, and significant stress and strain are also present in the contact area between the upper and lower parts, while the remaining areas experience relatively low stress and strain.

Based on the previous analysis, to reduce the shear strain on the coaxial surface of the fiber layer, the fiber layer should be arranged towards the outer circumference. However, the outer surface of the HWNR hose directly contacts the metal extrusion roller, and due to the adhesive friction and hysteresis friction between the contact surfaces, placing the fiber layer too close to the metal roller would exacerbate the separation between the NR matrix and the fiber layer. Therefore, selecting the maximum radius of the innermost layer of the fiber layer as 17.5mm reduces the shear strain on the coaxial surface of the hose, thereby increasing the service life of the composite material hose.

### Optimization design of fiber layer laying angle (LA)

For HWNR hoses, since the stiffness of the fiber layer material nylon is much greater than that of the rubber matrix, the main stress area is the fiber layer. Therefore, to increase the rigidity of the hose and simultaneously reduce the shear strain on the coaxial surface, we consider adjusting the fiber laying direction. Rewrite Eq. (2) in the form of a flexibility matrix:

5 $$ \left[ {\begin{array}{*{20}c} {\varepsilon_{x} } \\ {\varepsilon_{y} } \\ {\gamma_{xy} } \\ \end{array} } \right] = \left[ {\begin{array}{*{20}c} {\overline{S}_{11} } & {\overline{S}_{12} } & {\overline{S}_{16} } \\ {\overline{S}_{12} } & {\overline{S}_{22} } & {\overline{S}_{26} } \\ {\overline{S}_{16} } & {\overline{S}_{26} } & {\overline{S}_{66} } \\ \end{array} } \right]\left[ {\begin{array}{*{20}c} {\sigma_{x} } \\ {\sigma_{y} } \\ {\tau_{xy} } \\ \end{array} } \right] = \left[ {\begin{array}{*{20}c} {\frac{1}{{E_{x} }}} & {\frac{{ - \nu_{xy} }}{{E_{y} }}} & {\frac{{\eta_{x,xy} }}{{G_{xy} }}} \\ { - \frac{{\nu_{yx} }}{{E_{x} }}} & {\frac{1}{{E_{y} }}} & {\frac{{\eta_{y,xy} }}{{G_{xy} }}} \\ {\frac{{\eta_{xy,x} }}{{E_{x} }}} & {\frac{{\eta_{xy,y} }}{{E_{y} }}} & {\frac{1}{{G_{xy} }}} \\ \end{array} } \right]\left[ {\begin{array}{*{20}c} {\sigma_{x} } \\ \begin{gathered} \sigma_{y} \hfill \\ \tau_{xy} \hfill \\ \end{gathered} \\ \end{array} } \right] $$

where,

6 $$ \begin{array}{*{20}l} {\overline{S}_{11} = \frac{1}{{E_{x} }} = \frac{1}{{E_{1} }}\cos^{4} \theta + \left( {\frac{1}{{G_{12} }} - \frac{{2\nu_{21} }}{{E_{1} }}} \right)\sin^{2} \theta \cos^{2} \theta + \frac{1}{{E_{2} }}\sin^{4} \theta } \hfill \\ {\overline{S}_{22} = \frac{1}{{E_{y} }} = \frac{1}{{E_{1} }}\sin^{4} \theta + \left( {\frac{1}{{G_{12} }} - \frac{{2\nu_{{_{21} }} }}{{E_{1} }}} \right)\sin^{2} \theta \cos^{2} \theta + \frac{1}{{E_{2} }}\cos^{4} \theta } \hfill \\ \begin{gathered} \overline{S}_{12} = - \frac{{\nu_{yx} }}{{E_{x} }} = - \frac{{\nu_{21} }}{{E_{1} }}(\sin^{4} \theta + \cos^{4} \theta ) + \left( {\frac{1}{{E_{1} }} + \frac{1}{{E_{2} }} - \frac{1}{{G_{12} }}} \right)\sin^{2} \theta \cos^{2} \theta \\ \overline{S}_{66} = \frac{1}{{G_{xy} }} = \frac{1}{{G_{12} }}(\sin^{4} \theta + \cos^{4} \theta ) + 4(\frac{{1 + 2\nu_{21} }}{{E_{1} }} + \frac{1}{{E_{2} }} - \frac{1}{{2G_{12} }})\sin^{2} \theta \cos^{2} \theta \\ \overline{S}_{16} = \frac{{\eta_{xy,x} }}{{E_{x} }} = \left( {\frac{2}{{E_{1} }} + \frac{{2\nu_{21} }}{{E_{1} }} - \frac{1}{{G_{12} }}} \right){\text{sin}}\theta {\text{cos}}^{3} \theta - \left( {\frac{2}{{E_{2} }} + \frac{{2\nu_{21} }}{{E_{2} }} - \frac{1}{{G_{12} }}} \right){\text{sin}}^{3} \theta {\text{cos}}\theta \\ \overline{S}_{26} = \frac{{\eta_{xy,y} }}{{E_{y} }} = \left( {\frac{2}{{E_{1} }} + \frac{{2\nu_{21} }}{{E_{1} }} - \frac{1}{{G_{12} }}} \right){\text{sin}}^{3} \theta {\text{cos}}\theta - \left( {\frac{2}{{E_{2} }} + \frac{{2\nu_{21} }}{{E_{2} }} - \frac{1}{{G_{12} }}} \right){\text{sin}}\theta {\text{cos}}^{3} \theta \\ \end{gathered} \hfill \\ \end{array} $$

where *E* is the elastic modulus of the material in the principal elastic direction, $\nu$ is Poisson's ratio, *G* is the shear modulus in the plane, $\eta_{xy,x} ,\eta_{xy,y}$ and $\eta_{x,xy} ,\eta_{y,xy}$ are cross-elasticity coefficients, representing the ratios of $\gamma_{xy}$ to $\varepsilon_{x}$ when only $\sigma_{x}$ is present, of $\gamma_{xy}$ to $\varepsilon_{y}$ when only $\sigma_{y}$ is present, of $\varepsilon_{x}$ to $\gamma_{xy}$ when only $\tau_{xy}$ is present, and of $\varepsilon_{y}$ to $\gamma_{xy}$ when only $\tau_{xy}$ is present, respectively.

Introducing the dimensionless engineering elastic constants off-axis, *E*_*x*_/*E*_2_, *G*_*xy*_/*G*_12_, and *η*_*xy,x*_, into Eq. (6) yields Eq. (7).

7 $$ \begin{gathered} \frac{{E_{2} }}{{E_{x} }} = \frac{{E_{2} }}{{E_{1} }}\cos^{4} \theta + \left( {\frac{{E_{2} }}{{G_{12} }} - \frac{{2\nu_{21} E_{2} }}{{E_{1} }}} \right)\sin^{2} \theta \cos^{2} \theta + \sin^{4} \theta \\ \frac{{G_{12} }}{{G_{xy} }} = \sin^{4} \theta + \cos^{4} \theta + 4(\frac{{1 + 2\nu_{21} }}{{E_{1} }}G_{12} + \frac{{G_{12} }}{{E_{2} }} - \frac{1}{2})\sin^{2} \theta \cos^{2} \theta \\ \eta_{xy,x} = \left( {\frac{{2E_{x} }}{{E_{1} }} + \frac{{2\nu_{21} E_{x} }}{{E_{1} }} - \frac{{E_{x} }}{{G_{12} }}} \right){\text{sin}}\theta {\text{cos}}^{3} \theta - \left( {\frac{{2E_{x} }}{{E_{2} }} + \frac{{2\nu_{21} E_{x} }}{{E_{2} }} - \frac{{E_{x} }}{{G_{12} }}} \right){\text{sin}}^{3} \theta {\text{cos}}\theta \\ \end{gathered} $$

From Eq. (7), it can be observed that *E*_*x*_/*E*_2_ reaches its maximum value when *θ* = 0° and its minimum value when *θ* = 90°. *G*_*xy*_/*G*_12_ achieves its maximum value at 45°. *η*_*xy,x*_ is zero at 0° and 90°, with relatively large values at intermediate angles. Therefore, the design space is chosen around 45°. However, due to the complex deformation of the hose under compression, to obtain the optimal solution, multiple winding angles around the obtained angle are subjected to simulation analysis. The optimal design is selected based on the winding angle with the largest squeezing roller reaction force and the lowest shear strain within the coaxial surface. Considering practical production constraints, the selected range is from 35° to 70°.

### Optimization design of fiber layer laying gap (LG)

The spacing of the fiber layers also affects the performance of HWNR hoses. The Halpin–Tsai formula is used to calculate the modulus of elasticity for a single-layer board, as shown in Eq. (8)^10^:

8 $$ \begin{gathered} E_{1} \approx E_{f} c_{f} + E_{m} c_{m} \hfill \\ \nu_{21} = \nu_{f} c_{f} + \nu_{m} c_{m} \hfill \\ \frac{M}{{M_{m} }} = \frac{{1 + \zeta \eta c_{f} }}{{1 - \eta c_{f} }} \hfill \\ \end{gathered} $$

where, $\eta = \frac{{(M_{f} /M_{m} ) - 1}}{{(M_{f} /M_{m} ) + \zeta }}$, *c*_*f*_, and *c*_*m*_ represent the relative volume fractions of the fiber and matrix, where *c*_*f*_ + *c*_*m*_ = 1. *M* is the modulus of the composite material *E*_2_, *G*_12_, or *v*_32_; *M*_*f*_ corresponds to the modulus of the fiber *E*_*f*_, *G*_*f*_, or *v*_*f*_; Mm corresponds to the modulus of the matrix *E*_*m*_, *G*_*m*_, or *v*_*m*_. $\zeta$ is a measure of the fiber reinforcement effect related to fiber geometry, arrangement, and loading conditions.

It is evident that as the fiber volume fraction (*c*_*f*_) increases, the modulus of elasticity of the composite material increases, manifested by denser placement of nylon fibers and stronger elasticity of the composite material hoses. To achieve rubber hoses with higher stiffness, it is advisable to minimize the spacing between the fibers. Regarding the shear modulus, with an increase in the fiber volume fraction, the shear modulus of the composite material also increases. A higher shear modulus results in greater shear stiffness, thereby reducing the shear strain in the fiber layer. However, when the fiber volume fraction reaches a certain threshold, the shear modulus may tend to saturate because an increase in fibers may lead to increased interaction between them, thereby affecting the material's shear modulus. The Halpin–Tsai formula is mainly used to predict the transverse elastic modulus and is not accurate for predicting the shear modulus^31^, it can only roughly identify influencing factors. For the complex deformation process of extrusion rolling of HWNR hoses by the press roller, to obtain the optimal design, it is preferable to choose a smaller fiber layer spacing. Therefore, selecting a fiber layer spacing of 1–1.6 mm as the design space is recommended.

### Design space selection

Under the influence of major foreign manufacturers, the inner and outer diameters of HWNR hoses have become standardized, and the spacing between fiber layers in the hose has also stabilized. Changing these parameters would significantly increase production costs. The main parameters affecting the mechanical performance of the hose are the LG, LA and RP, as demonstrated in previous sections. For RP, the radius of the innermost fiber layer is selected as the design parameter, while keeping the interlayer spacing of the fiber layer unchanged at 1.5mm. Our objective is to obtain a hose with high elasticity, low shear strain on the fiber layer's coaxial surface, and long lifespan. These three factors interact with each other, making the situation complex under conditions of significant extrusion deformation. Based on the analysis presented earlier and considering actual production conditions, the design space is chosen as shown in Table 4.

**Table 4:** Optimization Design Space.

Factors	Design space
LG	1.0–1.6 mm
LA	35°–70°
RP	13.5–17.5 mm

## Parameter optimization and analysis

**Figure 9:**
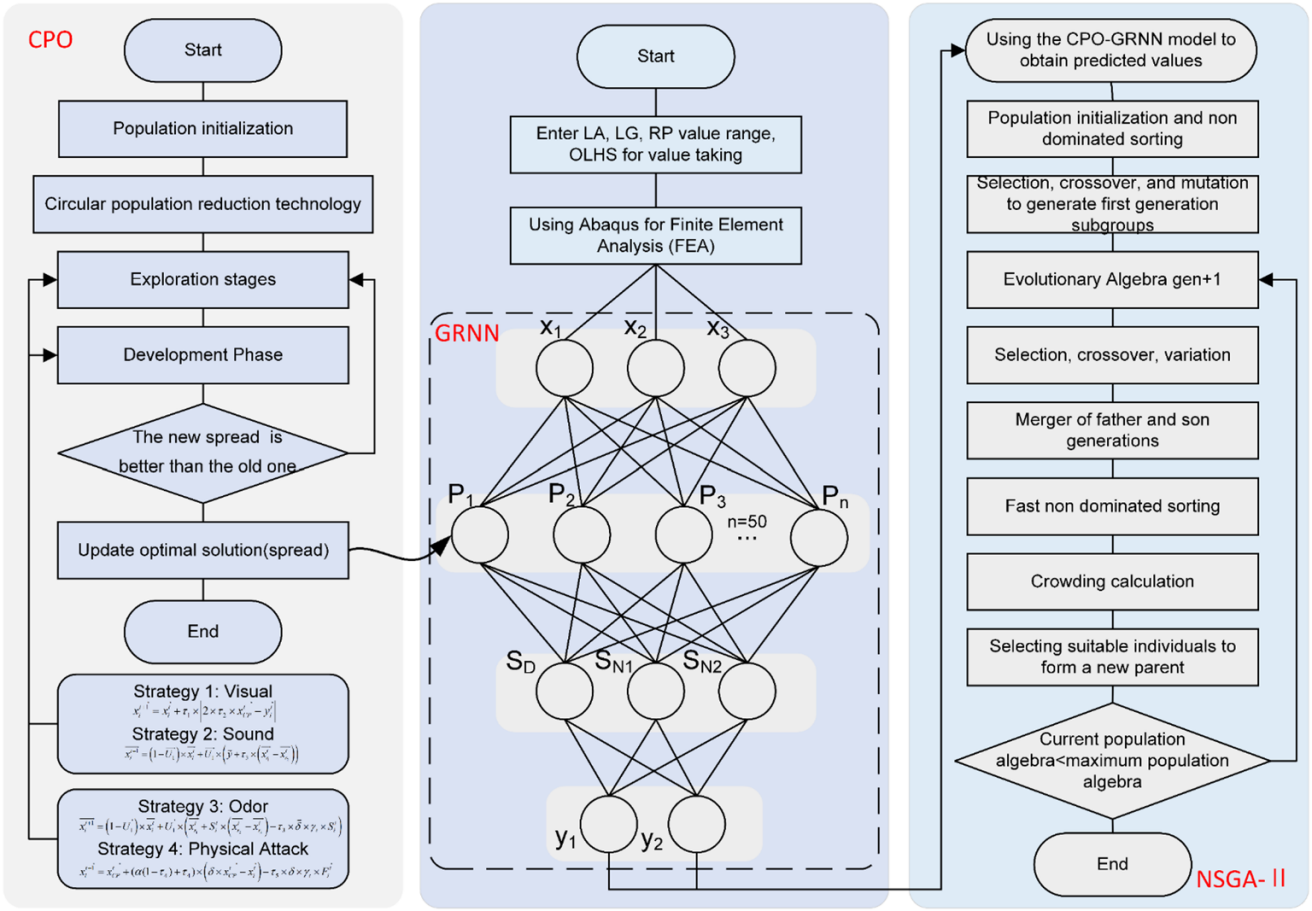
Flowchart of the CPO-GRNN-NSGA-II Algorithm.

Due to the complex stress conditions and numerous influencing factors affecting the mechanical performance of the HWNR hose during compression, and the strong nonlinearity involved, this paper employs neural networks instead of traditional RSM. Neural networks, in comparison, offer higher prediction accuracy for nonlinear problems. The optimization framework designed in this paper is illustrated in Fig. 9.

Firstly, by utilizing the Max–Min method to optimize the Latin Hypercube Sampling (LHS) method, we obtain an Optimal Latin Hypercube Sampling method (OLHS). The Max–Min method involves maximizing the minimum distance between each sample point and its preceding points, enhancing the dispersion of the sample points. Compared to LHS, orthogonal experiments, and response surface methodology, OLHS better fills the entire analysis space.

Subsequently, the CPO-GRNN was utilized for function approximation. GRNN, based on Radial Basis Function Neural Networks (RBFNN), inherits RBFNN's nonlinear mapping capability and fast learning speed. However, GRNN requires setting hyperparameters in advance, which significantly affect regression accuracy. CPO is utilized to find optimal hyperparameters, enhancing model accuracy. CPO is a metaheuristic population-based algorithm that simulates four defense mechanisms of the crested porcupine: visual, auditory, olfactory, and physical attacks. By dividing the predator's position into four regions corresponding to these strategies, CPO iteratively explores the optimal solution.

Contrary to traditional response surface methodology, which uses least squares to fit polynomial coefficients, neural network models bypass the process of function fitting, avoiding sequential experiments and exhibiting better nonlinear convergence for complex multifactorial experiments.

The NSGA-II algorithm with elite preservation strategy is finally employed for multi-objective optimization design. NSGA-II selects Pareto-optimal solutions and determines the optimal design parameters based on practical requirements.

### Establishment of surrogate models

Based on the description provided, the construction of the optimization objective function is as follows:

9 $$ \left\{ \begin{gathered} {\text{Max }}\left| {RF} \right| \hfill \\ {\text{Min }}\left| {MSS} \right| \hfill \\ s.t.\left\{ \begin{gathered} 1mm < LG < 1.6mm \hfill \\ 35^\circ < LA < 70^\circ \hfill \\ 13.5mm < RP < 17.5mm \hfill \\ \end{gathered} \right. \hfill \\ \end{gathered} \right. $$

Incorporate the constraints into OLHS to generate data. The neural network requires data at least ten times the number of variables. Select 50 sets of sample data for training and 10 sets for testing, as provided in the "Attachment".

Use the 50 sets of data to train CPO-GRNN. GRNN consists of four layers of neurons: the input layer, the pattern layer, the summation layer, and the output layer, as illustrated in Fig. 9. The pattern layer is similar to the RBFNN, where the output of neurons is the exponential square of the Euclidean distance between the input variables and their corresponding samples, as shown in Eq. (10). The number of neurons in the pattern layer equals the number of training samples.

10 $$ p_{i} = e^{{\left[ { - \frac{{\left( {X - X_{i} } \right)^{T} \left( {X - X_{i} } \right)}}{{2\delta^{2} }}} \right]}} ,i = 1,2, \cdots ,n $$

In the equation, *X* represents the test sample, *X*_*i*_ represents the training sample, and $\delta$ is the hyperparameter of the model, whose value is related to the smoothness of the Gaussian function.

The summation layer consists of two parts: *S*_*D*_, which is the arithmetic sum of the outputs from the pattern layer; and *S*_*N*_, which is the weighted sum of the outputs from the pattern layer. The calculation formula is shown as follows:

11 $$ \begin{gathered} S_{D} = \mathop \sum \limits_{i = 1}^{m} g_{i} \hfill \\ S_{Nj} = \mathop \sum \limits_{i = 1}^{m} y_{ij} g_{i} ,j = 1,2, \cdots ,k \hfill \\ \end{gathered} $$

where the weighting coefficient *y*_*ij*_ corresponds to the j-th element of the label of the training sample associated with the j-th pattern layer node.

The last layer is the output layer, with the number of nodes equal to the dimension of the label vector. The output of each node is equal to the corresponding sum layer output divided by the output of the first node of the sum layer. The calculation Eq. (12) is shown below.

12 $$ y_{j} = \frac{{S_{Nj} }}{{S_{D} }},j = 1,2, \cdots ,k $$

In MATLAB, the GRNN function has three input parameters, with the third parameter, spread, determined by the hyperparameter $\delta$,${\text{spread = }}\frac{{\sigma^{2} }}{1.6652}$. The selection of this coefficient affects the smoothness of the function approximation. Choosing a reasonable spread parameter is helpful in improving the accuracy of the regression model. Using CPO to select the spread parameter.

The CPO algorithm demonstrates good stability and robustness in complex optimization problems due to its simplicity, wide exploration of the search space, maintenance of population diversity, acceleration of convergence speed, and avoidance of local minima. The CPO algorithm first initializes the function, using the Circular Population Reduction (CPR) technique, which not only accelerates convergence but also maintains population diversity. Following that, through a four-layer defense mechanism, the population is continuously optimized to explore the optimal value. The mathematical expressions for the four-layer defense mechanisms of vision, sound, smell, and physical attacks are respectively:

13 $$ \begin{gathered} \overrightarrow {{x_{i}^{t + 1} }} = \overrightarrow {{x_{i}^{t} }} + \tau_{1} \times \left| {2 \times \tau_{2} \times \overrightarrow {{x_{CP}^{t} }} - \overrightarrow {{y_{i}^{t} }} } \right| \hfill \\ \overrightarrow {{x_{i}^{t + 1} }} = \left( {1 - \overrightarrow {{U_{1} }} } \right) \times \overrightarrow {{x_{i}^{t} }} + \overrightarrow {{U_{1} }} \times \left( {\vec{y} + \tau_{3} \times \left( {\overrightarrow {{x_{{r_{1} }}^{t} }} - \overrightarrow {{x_{{r_{2} }}^{t} }} } \right)} \right) \hfill \\ \begin{array}{*{20}c} {\overrightarrow {{x_{i}^{t + 1} }} = \left( {1 - \overrightarrow {{U_{1} }} } \right) \times \overrightarrow {{x_{i}^{t} }} + \overrightarrow {{U_{1} }} \times \left( {\overrightarrow {{x_{{r_{1} }}^{t} }} + S_{i}^{t} \times \left( {\overrightarrow {{x_{{r_{2} }}^{t} }} - \overrightarrow {{x_{{r_{3} }}^{t} }} } \right) - \tau_{3} \times \vec{\delta } \times \gamma_{t} \times S_{i}^{t} } \right)} \\ \end{array} \hfill \\ \overrightarrow {{x_{i}^{t + 1} }} = \overrightarrow {{x_{CP}^{t} }} + (\alpha (1 - \tau_{4} ) + \tau_{4} ) \times \left( {\delta \times \overrightarrow {{x_{CP}^{t} }} - \overrightarrow {{x_{i}^{t} }} } \right) - \tau_{5} \times \delta \times \gamma_{t} \times \overrightarrow {{F_{i}^{t} }} \hfill \\ \end{gathered} $$

In the equation, $\overrightarrow {{x_{i}^{t + 1} }}$ represents the updated CP position; where $\overrightarrow {{x_{CP}^{t} }}$ is the best solution evaluated by the function at iteration *t*; $\overrightarrow {{y_{i}^{t} }}$ is the position of the predator at iteration *t*; $\tau_{1}$ is a random number generated based on a normal distribution; $\tau_{2} ,\tau_{3} ,\tau_{4}$ are random values generated between [0,1]; $r_{1} ,r_{2}$ are random integers generated between [1, N]; $r_{3}$ is a random number generated between [1, N]; $\overrightarrow {{U_{1} }}$ is a binary vector randomly generated between [0,1], used to represent the impact of CP sound and odor factors on the predator; $S_{i}^{t}$ is used to optimize the odor diffusion rate; $\vec{\delta }$ is used to control the search direction; $\gamma_{t}$ is a defined defense factor; $\overrightarrow {{F_{i}^{t} }}$ is the average force of the CP affecting the i-th predator; $\alpha$ is the convergence rate factor.

**Figure 10:**
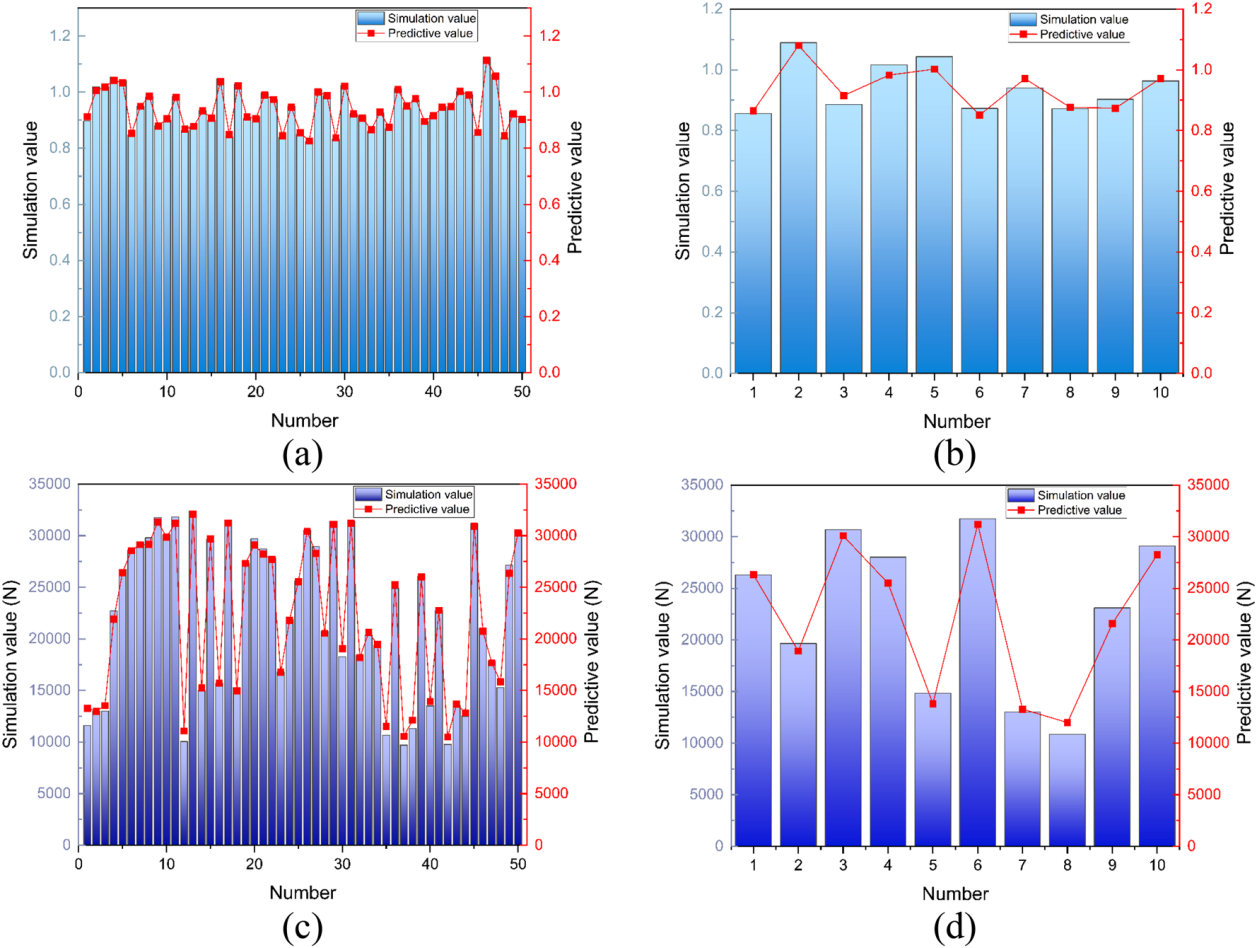
Training results. (**a**) Comparison of MSS training values and simulation values. (**b**) Comparison of MSS test values and simulation values. (**c**) Comparison of RF training values and simulation values. (**d**) Comparison of RF test values and simulation values.

CPO-GRNN inherits the advantages of GRNN. When the data volume is small, it still exhibits good accuracy in its predictions. Additionally, because the network is trained in a single pass without the need for iteration, it operates at a faster speed. According to the mathematical principles of CPO-GRNN, the activation function of the hidden layer nodes adopts a Gaussian function with local activation characteristics for input information. This makes it highly attractive to inputs that closely resemble the characteristics of local neurons. Moreover, the CPO algorithm, which has a wide search space and fast convergence speed, is utilized. Therefore, CPO-GRNN possesses strong function approximation capabilities. Using the 50 sets of data selected by OLHS as training samples, with LA, LG, and RP as input data and MSS and RF as output data, they are imported into CPO-GRNN for training. The training results are shown in Fig. 10.

Compare the trained model with the traditional RSM. For RSM, the commonly used Box-Behnken Design (BBD) model, similar to systems, is employed^32–36^, selecting three factors and a five-center point design. The design space is shown in Table 5.

**Table 5:** Parameters of RSM Design Space.

Number	LG (mm)	LA (°)	RP (mm)	MSS	RF (N)
1	1.3	52.5	15.5	0.938772	− 24,117.8
2	1.3	52.5	15.5	0.938772	− 24,117.8
3	1	35	15.5	0.849433	− 32,302.9
4	1	52.5	13.5	1.1205	− 21,003
5	1.6	52.5	17.5	0.863148	− 24,286.9
6	1.3	70	13.5	0.946	− 9839
7	1.6	52.5	13.5	1.0156	− 23,404
8	1	52.5	17.5	0.873052	− 25,388.6
9	1	70	15.5	0.954232	− 9331.5
10	1.3	35	13.5	0.9127	− 29,593
11	1.3	70	17.5	0.832088	− 8959.46
12	1.6	35	15.5	0.840975	− 30,727.9
13	1.3	52.5	15.5	0.938772	− 24,117.8
14	1.3	52.5	15.5	0.938772	− 24,117.8
15	1.3	52.5	15.5	0.938772	− 24,117.8
16	1.3	35	17.5	0.806492	− 30,483.7
17	1.6	70	15.5	0.935157	− 9295.23

Through the experimental design process, the response surface regression equations can be obtained as shown in Eqs. (14) and (15).

14 $$ \begin{gathered} Y_{MSS} = 2.34829 - 1.36752X_{LG} + 0.026937X_{LA} - 0.120929X_{RP} - 0.000506X_{LG} X_{LA} + 0.039582X_{LG} X_{RP} \\ - 0.000055X_{LA} X_{RP} + 0.277401X_{LG}^{2} - 0.000225X_{LA}^{2} + 0.001084X_{RP}^{2} \\ \end{gathered} $$

15 $$ \begin{gathered} Y_{RF} = 55066.78280 - 17223.20556X_{LG} - 775.33436X_{LA} - 7900.58865X_{RP} - 73.27286X_{LG} X_{LA} \\ + 1459.45833X_{LG} X_{RP} + 12.64457X_{LA} X_{RP} - 546.76389X_{LG}^{2} + 12.25347X_{LA}^{2} + 161.59594X_{RP}^{2} \\ \end{gathered} $$

where, *X*_*LA*_, *X*_*LG*_, *X*_*RP*_ represent the levels of LA, LG, and RP factors respectively, while *Y*_*MSS*_ and *Y*_*RF*_ represent the values of MSS and RF.

Using Mean Absolute Error (MAE), Mean Squared Error (MSE), Root Mean Squared Error (RMSE), Mean Absolute Percentage Error (MAPE), and R-squared (R^2^) as measures, five evaluation criteria for the models can avoid errors offsetting each other and accurately reflect the actual prediction errors^37^.

Comparing the CPO-GRNN model with RSM, as shown in Table 6, it is evident that the CPO-GRNN model exhibits significantly higher prediction accuracy than RSM. For MSS, compared to RSM, the CPO-GRNN model shows a reduction of 38.88% in MAE, 69.69% in MSE, 44.95% in RMSE, 37.55% in MAPE, and an improvement of 36.18% in R^2^. Regarding RF, compared to RSM, the CPO-GRNN model demonstrates a reduction of 10.56% in MAE, 13.75% in MSE, 7.13% in RMSE, 20.82% in MAPE, and a 0.4% increase in R^2^. Therefore, the prediction accuracy of CPO-GRNN is significantly higher than that of RSM. Using CPO-GRNN for predicting the mechanical performance of HWNR hoses enhances the optimization accuracy.

**Table 6:** Error Analysis of CPO-GRNN Model and RSM Model.

	CPO-GRNN		RSM	
	MSS	RF	MSS	RF
MAE	0.0215	924.395	0.0352	1033.553
MSE	0.000616	1,309,070.682	0.00203	1,517,676.241
RMSE	0.0248	1144.146	0.0451	1231.94
MAPE	2.268%	-4.565%	3.632%	-5.765%
R^2^	0.938	0.976	0.643	0.972

### Optimization results

**Figure 11:**
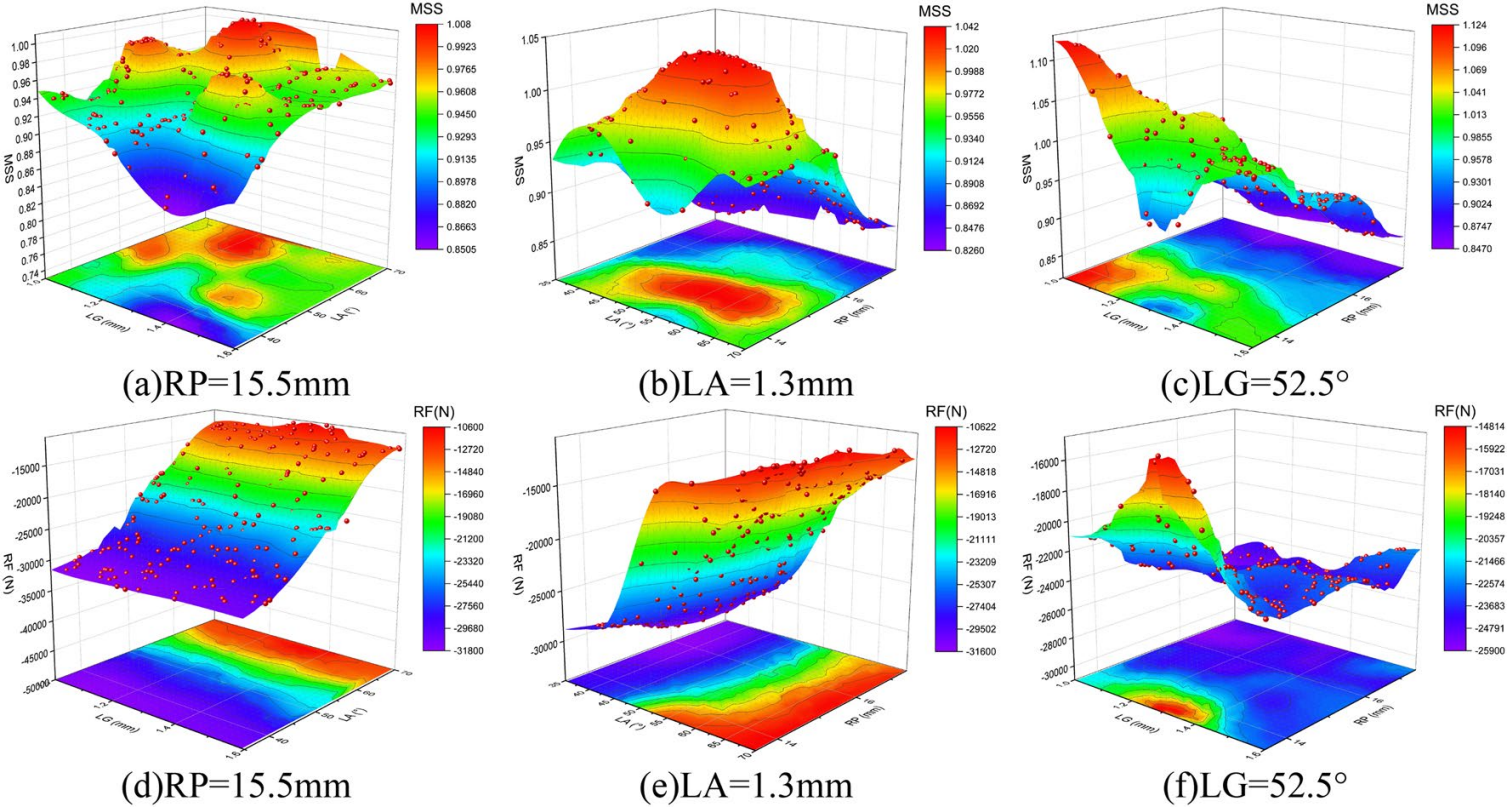
The response surface plots are generated through scatter point fitting based on CPO-GRNN. (**a**)–(**c**) are the MSS response surfaces. (**d**)–(**f**) are the RF response surfaces.

To more intuitively observe the effects of fiber layer arrangement parameters on MSS and RF, response surfaces generated based on CPO-GRNN were obtained, as shown in Fig. 11. These surfaces illustrate the strong nonlinearity of the function and the influence trends of various factors on MSS and RF.

**Figure 12:**
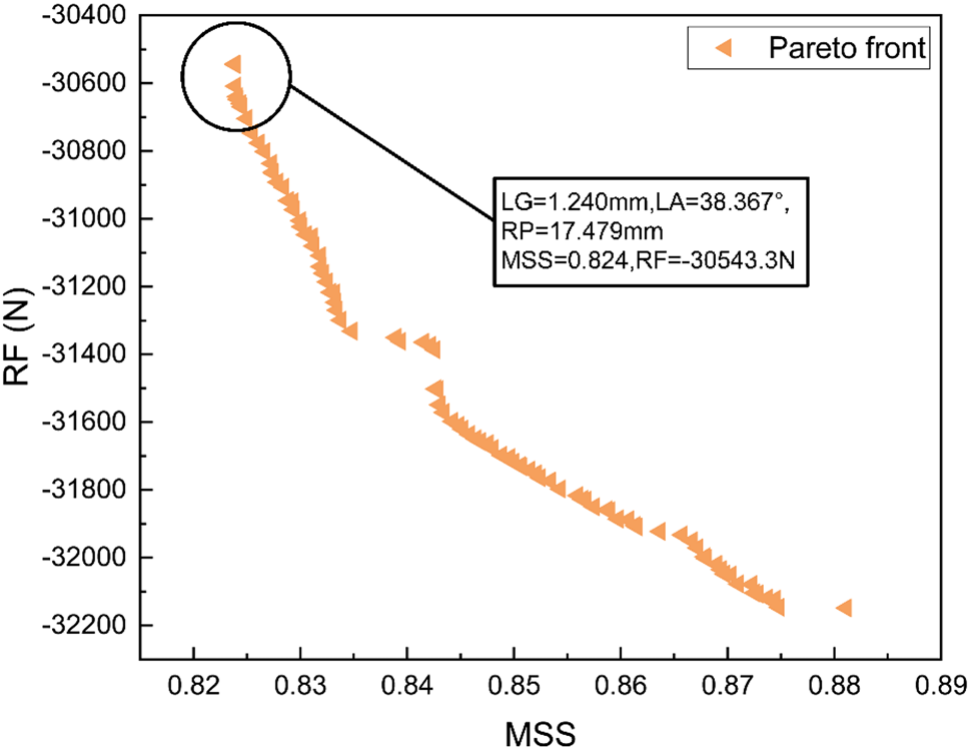
Pareto Front Obtained Through Optimization.

To obtain the minimum absolute value of MSS and the maximum absolute value of RF, the trained model is imported into NSGA-II. NSGA-II is a mature multi-objective optimization algorithm. The algorithm iterates the population using genetic algorithms, ranks candidate solutions using non-dominated sorting to determine which solutions are more optimal, maintains population diversity by calculating crowding distances, and encourages a uniform distribution of solutions within the Pareto optimal solution set. Through these operations, a Pareto frontier is selected, yielding a series of optimal solutions, as shown in Fig. 12.

**Figure 13:**
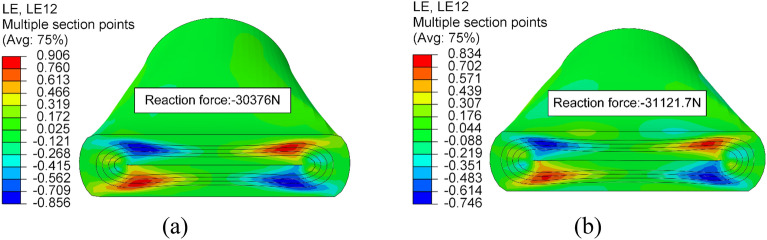
Presents the shear strain contour plots and reaction force of the optimized results and the original HWNR hose when the squeezing roller is pressed down by 35 mm. In (**a**), the shear strain contour plot and reaction force of the original HWNR hose are depicted, while (**b**) displays the shear strain contour plot and reaction force of the optimized HWNR hose.

Compared to the influence of MSS on the fatigue life of HWNR hoses, the impact of rebound stiffness on the fluid transport efficiency of HWNR hoses is minimal. Therefore, we select the point with the minimum MSS in the Pareto frontier as the final result of the optimization design. At this point, LG = 1.24mm, LA = 38.367°, and RP = 17.479 mm, as shown in Fig. 12. FEA is utilized to simulate the optimized results. A comparison between the simulated values and predicted values reveals an error of 1.22% for MSS and 1.86% for RF, indicating a high level of confidence in the results. A comparison between the optimized HWNR hose and the original HWNR hose is illustrated in Fig. 13. The new hose shows a reduction of 7.99% in MSS and an increase of 2.46% in RF. Thus, while enhancing the service life of the hose, the new hose also improves fluid transport capability.

## Concluding remarks

This study, starting from engineering practicality, aims to obtain HWNR hoses with enhanced liquid fertilizer transport capacity and stronger fatigue resistance. The CPO-GRNN-NSGA-II method is employed for comprehensive optimization design of the mechanical properties of HWNR hoses.

Initially, a finite element simulation model of HWNR hoses is established using the dispersion method to arrange reinforcement films, and the accuracy of this model is verified through experiments within a 5% margin.

Subsequently, the primary cause of fatigue failure was determined to be MSS. a mathematical model of HWNR hose materials is developed using laminate theory and the Halpin–Tsai formula. The effects of LG, LA, and RP on the optimization objectives of MSS and RF are analyzed, and the design space is determined based on actual production conditions.

For the first time, the CPO-GRNN method is utilized to establish a surrogate model for predicting the mechanical properties of HWNR hoses. Its accuracy is significantly higher than that of RSM. Regarding the prediction of MSS, compared to RSM, the CPO-GRNN model reduces MAE, MSE, RMSE, and MAPE by 38.88%, 69.69%, 44.95%, and 37.55%, respectively, and increases R^2^ by 36.18%. For RF prediction, compared to RSM, the CPO-GRNN model reduces MAE, MSE, RMSE, and MAPE by 10.56%, 13.75%, 7.13%, and 20.82%, respectively, and increases R^2^ by 0.4%.

Finally, NSGA-II is used for multi-objective optimization design to obtain the Pareto frontier. FEA is performed for validation, resulting in a 1.22% error between the predicted and simulated values for MSS, and a 1.86% error for RF. LG = 1.24 mm, LA = 38.367°, and RP = 17.479 mm are chosen as the final optimized design parameters. This design reduces MSS by 7.99% and increases RF by 2.46%, significantly enhancing both the service life and liquid fertilizer transport efficiency of the HWNR hoses.

## Supplementary Information

Supplementary Information.

## Data Availability

The datasets used or analyzed during the current study are available from the corresponding author on reasonable request.

## Code availability

The numerical model software during the current study are available from the corresponding author on reasonable request.
