# Supplementary material for: Prediction and design optimization of mechanical properties for rubber fertilizer hose reinforced with helically wrapped nylon
Source: Sci Rep. 2024 Jun 10;14:13261. doi: 10.1038/s41598-024-64233-y (PMC11637102; doi:10.1038/s41598-024-64233-y)
Supplement: Supplementary file 1 — Supplementary Information. [file 41598_2024_64233_MOESM1_ESM.docx]

**Attachment**

Training set

| Number | LG(mm) | LA(°) | RP(mm) | MSS | RF(N) |
| --- | --- | --- | --- | --- | --- |
| 1 | 1.31 | 55.26 | 13.6 | 0.897219 | -11585.1 |
| 2 | 1.2 | 66.17 | 15.71 | 1.01884 | -13001.2 |
| 3 | 1.02 | 63.69 | 14.33 | 1.01884 | -13001.2 |
| 4 | 1.3 | 52.86 | 14.63 | 1.05442 | -22719.7 |
| 5 | 1.08 | 49.2 | 14.88 | 1.04134 | -26715.3 |
| 6 | 1.39 | 46.56 | 17.38 | 0.848229 | -28619.9 |
| 7 | 1.41 | 38.75 | 14.43 | 0.94856 | -29359 |
| 8 | 1.11 | 42.43 | 13.96 | 0.9839 | -29791 |
| 9 | 1.28 | 39.26 | 16 | 0.880221 | -31755.5 |
| 10 | 1.03 | 44.93 | 15.85 | 0.899939 | -30125.9 |
| 11 | 1.04 | 40.17 | 14.79 | 0.983395 | -31828.6 |
| 12 | 1.13 | 68.83 | 16.71 | 0.862317 | -10046.2 |
| 13 | 1.12 | 36.1 | 16.29 | 0.878187 | -32385.8 |
| 14 | 1.06 | 62.79 | 16.07 | 0.934226 | -14958.3 |
| 15 | 1.22 | 35.5 | 14.27 | 0.900157 | -29646.9 |
| 16 | 1.29 | 61.36 | 14.77 | 1.04701 | -15678 |
| 17 | 1.07 | 41.4 | 17.02 | 0.84188 | -31529 |
| 18 | 1.44 | 60.16 | 14.01 | 1.02785 | -15271.8 |
| 19 | 1.24 | 47.92 | 15.64 | 0.905335 | -27302.5 |
| 20 | 1.14 | 47.13 | 16.52 | 0.91389 | -29694.5 |
| 21 | 1.42 | 45.72 | 15.11 | 0.998343 | -28703 |
| 22 | 1.33 | 42.91 | 13.68 | 0.9733 | -27843 |
| 23 | 1.09 | 60.59 | 17.16 | 0.839207 | -16802.6 |
| 24 | 1.16 | 55.68 | 15.96 | 0.949587 | -21900.1 |
| 25 | 1.21 | 51.37 | 17.29 | 0.850603 | -25602.9 |
| 26 | 1.23 | 40.63 | 17.23 | 0.822714 | -30520.4 |
| 27 | 1.25 | 44.47 | 14.57 | 1.00791 | -28945.6 |
| 28 | 1 | 56.56 | 15.29 | 0.986811 | -20698.7 |
| 29 | 1.38 | 38.23 | 16.9 | 0.832987 | -31419.8 |
| 30 | 1.18 | 50.43 | 13.57 | 1.023 | -18251 |
| 31 | 1.17 | 37.63 | 15.25 | 0.923504 | -31415.7 |
| 32 | 1.27 | 59.38 | 16.61 | 0.914774 | -18406.6 |
| 33 | 1.43 | 56.93 | 17.01 | 0.861107 | -20375.5 |
| 34 | 1.37 | 57.45 | 15.76 | 0.92708 | -19378.1 |
| 35 | 1.35 | 67.73 | 16.63 | 0.870549 | -10652.8 |
| 36 | 1.46 | 48.4 | 14.2 | 1.01271 | -24893.1 |
| 37 | 1.32 | 69.31 | 15.36 | 0.943447 | -9702.56 |
| 38 | 1.1 | 66.95 | 15.02 | 0.970749 | -11309.6 |
| 39 | 1.01 | 52.02 | 16.78 | 0.896223 | -26073 |
| 40 | 1.47 | 64.05 | 16.14 | 0.91678 | -13493.9 |
| 41 | 1.51 | 53.59 | 15.47 | 0.945137 | -22756.2 |
| 42 | 1.34 | 68.44 | 14.07 | 0.943229 | -9764.71 |
| 43 | 1.19 | 65.69 | 13.78 | 1.0017 | -13560 |
| 44 | 1.45 | 64.99 | 14.96 | 0.99194 | -12507.7 |
| 45 | 1.4 | 36.85 | 15.54 | 0.846067 | -31150.1 |
| 46 | 1.05 | 54 | 13.87 | 1.1242 | -21026.5 |
| 47 | 1.15 | 58.35 | 14.49 | 1.06201 | -17850.9 |
| 48 | 1.26 | 62.07 | 17.48 | 0.836944 | -15289.4 |
| 49 | 1.36 | 50.21 | 16.45 | 0.931159 | -27134.7 |
| 50 | 1.48 | 43.64 | 16.37 | 0.905802 | -30495.8 |

Test set

| Number | LG(mm) | LA(°) | RP(mm) | MSS | RF(N) |
| --- | --- | --- | --- | --- | --- |
| 1 | 1.18 | 50.64 | 17.11 | 0.856447 | -26297.9 |
| 2 | 1.12 | 56.77 | 13.7 | 1.0889 | -19652 |
| 3 | 1.02 | 43.58 | 15.62 | 0.886071 | -30699.2 |
| 4 | 1.57 | 46.11 | 14.91 | 1.01639 | -28023.4 |
| 5 | 1.45 | 61.98 | 14.66 | 1.04309 | -14828.7 |
| 6 | 1.4 | 36.01 | 16.14 | 0.872629 | -31726.9 |
| 7 | 1.09 | 64.6 | 15.37 | 0.940407 | -13015.3 |
| 8 | 1.31 | 67.62 | 16.65 | 0.871417 | -10833.7 |
| 9 | 1.49 | 54.33 | 16.71 | 0.902847 | -23107.9 |
| 10 | 1.29 | 41.36 | 13.94 | 0.9633 | -29088 |
